# Honey Quality Control: Review of Methodologies for Determining Entomological Origin

**DOI:** 10.3390/molecules28104232

**Published:** 2023-05-22

**Authors:** Saeed Mohamadzade Namin, Sampat Ghosh, Chuleui Jung

**Affiliations:** 1 Agricultural Science and Technology Institute, Andong National University, Andong 36729, Republic of Korea; 2 Department of Plant Medicals, Andong National University, Andong 36729, Republic of Korea

**Keywords:** honey, adulteration, DNA, SDS-PAGE, DNA metabarcoding, chemical profiling, entomological origin

## Abstract

Honey is a widely consumed natural product, and its entomological origin can significantly influence its market value. Therefore, traceability of the entomological origin of honey should also be considered in honey quality control protocols. Although several methods exist, such as physicochemical characterization and bioactivity profiling of honey of different entomological origins, the most promising three methods for entomological authentication of honey include protein-based identification, chemical profiling, and a DNA-based method. All of these methods can be applied for reliable identification of the entomological origin of honey. However, as the honey is a complex matrix, the inconsistency of the results obtained by these methods is a pragmatic challenge, and therefore, the use of each method in all the cases is questionable. Most of these methodologies can be used for authentication of newly harvested honey and it is worth understanding the possibility of using these methods for authentication of relatively old samples. Most probably, using DNA-based methods targeting small fragments of DNA can provide the best result in old samples, however, the species-specific primers targeting short fragments are limited and not available for all species. Therefore, using universal primers in combination with a DNA metabarcoding approach can be a good solution that requires further investigation. This present article describes the applications of different methods, their pros, and their cons to identify honey based on entomological origin.

## 1. Introduction

Honey is a sweet natural product that is produced by managed and wild bees, derived from the nectar of flowers. It is made up of various components such as sugar, protein, vitamins, minerals, aromatic substances, polyphenols, pigments, beeswax, and pollen that contribute to its color, smell, and flavor [1,2]. However, honey adulteration is a growing concern, including the production of honey by feeding bees with commercial industrial sugar [3], the addition of foreign sugar, as well as mislabeling. The botanical, geographical, and entomological origin of honey greatly influences its price [4,5,6,7,8,9,10]. Among honeybees, the European honeybee, *Apis mellifera*, and the Eastern honeybee, *A. cerana*, are two dominant species for honey production [11]. 

*Apis cerana* is native to temperate and tropical Asia and is widespread from Afghanistan to far east Russia and Japan, north into the foothills of the Himalayan mountains, and south through Indonesia [12,13]. This species was introduced to New Guinea in the late 1970s from Indonesia and became invasive to Australia in 1998 [14], and eradication attempts against it remain unsuccessful [14,15,16]. European honeybee, *A. mellifera*, is native to Europe, the Middle East, and Africa, and was introduced to other continents in the 17th century. It is now widespread around the world [17,18]. Because *A. mellifera* is more productive and suitable for modern beekeeping, it has been introduced into many *A. cerana* habitats, such as China, South Korea, and some Southeast Asian countries [19,20]. Afterwards, the number of *A. cerana* colonies declined due to high interspecific competition for the same niche as *A. mellifera*. Other than managed honeybees, honey can be harvested from the colonies of wild *Apis* bees and stingless bees. As a result of recent discoveries about the benefits of honey consumption, the demand for natural honey has increased and the price of natural honey is five to seven times higher than *A. mellifera* honey [8,11]. Therefore, honey from *A. cerana* and wild bees is very prone to adulteration, either by mislabeling or mixing the natural honey with cheaper *A. mellifera* honey to gain a higher profit [8,11]. 

In addition, honey in Europe is produced by *A. mellifera*. However, based on morphological [12] and molecular analyses [21], about ten subspecies of *A. mellifera* are present in Europe, belonging to three different lineages: The African (A), the Eastern European (C), and the Western European (M). Understanding the lineage of honeybee from which the honey originates is helpful in identifying the geographical origin of the honey, which is necessary for quality control assessment of honey samples. While various methodologies are used to identify the entomological origin of honey, a comprehensive review of these methods is currently unavailable. Hence, the objective of this review is to provide a summary of various methodologies used to identify the entomological origin of honey, assess their respective advantages and disadvantages, and help researchers choose the most appropriate method for their sample identification. Furthermore, this review may offer valuable insights into the development of novel methodologies. 

## 2. Identification Methods of Entomological Origin of Honey

### 2.1. Protein Based Method 

**Figure 1:**
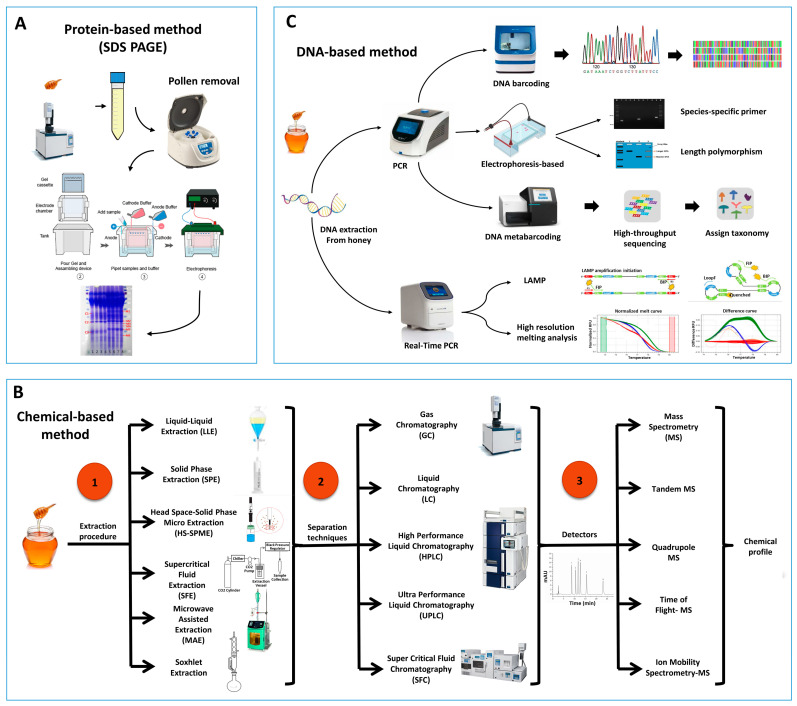
Schematic representation of the commonly used sample processing and techniques for the study of the entomological origin of honey. (**A**) Protein-based method, (**B**) Chemical-based methods emphasizing on different extraction methods, separation techniques, and Detection of identification of compounds, (**C**) DNA-based methods.

Sodium Dodecyl Sulfate Polyacrylamide Gel Electrophoresis (SDS-PAGE) is a popular technique for separating proteins based on their mass. In an electric field, proteins migrate through the gel from the negatively charged electrode toward the positively charged electrode, allowing for separation of proteins based on molecular weight. SDS-PAGE, as depicted in Figure 1A, has frequently been used to identify the entomological origin of honey from both managed honeybees and stingless bees [22,23]. Despite the limited amount of protein in honey, some studies have been conducted on its protein profile using methods such as Matrix-Assisted Laser Desorption Ionization Time of Flight Mass Spectrometry (MALDI-TOF-MS) and Liquid Chromatography Mass Spectrometry (LC-MS/MS) [1,24,25]. However, in the majority of cases, these studies have not specifically focused on distinguishing the entomological source of honey. The Major Royal Jelly Proteins (MRJPs) are a group of nine homologous proteins (MRJP1–MRJP9) [26,27,28] secreted by worker bees. Along with pollen proteins, they are significant protein resources in honey [1,29]. As the MRJPs of different bee species are specific and they remain in the honey, there is a possibility of using such a protein to identify the entomological origin of honey. Previous studies have shown that the protein profile of SDS-PAGE is different between honey samples from *A. cerana* and *A. mellifera* [22]. Moreover, Won et al. [1] showed that MRJP1 has different molecular weights in different honeybees (56 kDa in *A. cerana* and 59 kDa in *A. mellifera*) despite having similar primary structure. By producing an artificial marker protein from *Escherichia coli* and co-electrophoresing it with honey samples, they successfully discriminated the entomological origin of honey samples by SDS–PAGE and found several adulterated honey samples. Additionally, Zhang et al. [30] indicated that three species-specific bands with molecular weight between 15.0 and 29.4 KDa are present in *A. cerana* honey, while *A. mellifera* honey is characterized by the appearance of six species-specific bands with molecular weights between 13.8 and 33.1 KDa. 

### 2.2. Physicochemical Properties and Bioactive Characterization of Honey to Determine Entomological Origin

The chemical properties of honey have been used to identify the entomological origin of honey. Zannat et al. [31] showed that the physicochemical profile of honey such as moisture, formaldehyde and Hydroxymethylfurfural (HMF), pH, and color were key determinants to identify the entomological origin of Bangladesh honey samples, such as *A. cerana*, *A. dorsata*, and *A. mellifera* honey. This method can be applied to detect the mislabeling of honey. Furthermore, it seems that Principle Component Analysis (PCA) involving the physicochemical properties of honey can be useful for identification of the entomological origin of honey samples regionally, but the results cannot always be extended to other regions due to the high variation in the chemical properties of honey from different localities. For instance, the moisture content of honey samples from Bangladesh were 21.3, 19, and 17% from *A. dorsata*, *A. cerana* and *A. mellifera*, respectively. However, the reported amount of moisture for *A. cerana* honey from Thailand was 21.2% [32] which is comparable with the amount of moisture in *A. dorsata* honey from Bangladesh. Furthermore, moisture content of *A. mellifera* honey from Thailand was reported as 18.8%, which was similar to the amount of moisture in *A. cerana* honey from Bangladesh. On the other hand, using the color of honey as a method of recognition cannot be recommended due to its variation based on the floral source of honey, storage duration, and storage conditions. However, since multidimensional analysis, such as PCA, is required for categorization of honey samples with different origins, more studies are required to understand the possibility of the usage of this method in identification of the entomological origin of honey. 

Another attempt to identify the entomological origin of honey is bioactive characterization [33,34,35,36] and mineral content [37,38]. Kelulut honey, produced by stingless bees *Heterotrigona itama*, was found distinguishable from *Apis* spp. honey based on characteristics such as high moisture, free acidity, color intensity, and antioxidant capacities measured by AEAC and FRAP assays [34]. Findings of the study by Wu et al. [36] illustrated that *Lepidotrigona flavibasis* honey exhibited the highest antioxidant activity (in terms of DPPH and FRAP assay), proline content, flavonoid content, and phenolic content among *A. cerana cerana*, *A. dorsata,* and *L. flavibasis* honey samples. Rodríguez-Malavera et al. [33] characterized 16 honey samples from 10 stingless bee species (*Melipona crinita*, *M. eburnea*, *M. grandis*, *M. illota*, *Nannotrigona melanocera*, *Partamona epiphytophila*, *Ptilotrigona lurida*, *Scaptotrigona polystica*, *Scaura latitarsis*, and *Tetragonisca angustula*) based on physicochemical properties (color and moisture), biochemical components (such as flavonoid, polyphenol, nitrites and protein contents), and bioactive properties (including antibacterial and antioxidant activities). Although the Trolox Equivalent Antioxidant Capacities (TEAC) measured by decoloration of the ABTS + ● radical cation were found to be different for honey samples from different bee species, the sample number of individual honey types was quite limited [33]. Biluca et al. [39] demonstrated the difference in moisture content and acidity on the 35 honey samples from stingless bees in Brazil compared to values of *Apis mellifera*. Although the study indicated higher antioxidant activities and phenolic contents of the stingless bee honey, there was no specific marker for identifying its entomological origin [39]. A review by Ávila et al. [35] also demonstrated the broader biological activities of stingless bees compared to *Apis mellifera* honey. Kek et al. [37] indicated the possibility of distinguishing honey from different bee species such as *Apis* spp. and *Heterotrigona* spp. based chemical composition and mineral contents. However, although honey of different bee species is characterized with different physicochemical properties, bioactive characteristics, and minerals profile, it is often influenced by geographical location, floral origin, and storage conditions.

### 2.3. Chemical Profiling to Authenticate Entomological Origin of Honey

Due to intricate chemical makeup of honey with variations of physicochemical properties, integration of chemical analyses with compound profiling are employed in scientific studies on the authentication of honey. Figure 1B represents the typical approach of sample processing and chromatographic techniques used widely in scientific studies. 

The analysis of volatile compounds is an alternative and promising approach in the authentication of honey samples of different entomological origin. Volatile compounds have a high vapor pressure at room temperature, and therefore, easily evaporate and turn into gas. Besides contributing to the unique fragrance, flower volatiles play vital role in attracting pollinators. Several studies have indicated that these volatile compounds and metabolites remain consistent in flowers, nectar, or honeydew which can be traced back to the specific floral source of the honey. For instance, 2-methoxybenzoic acid and 3-phenyllactic acid are biomarkers for Manuka honey, and methyl anthranilate is a marker for citrus honey [38,40]. Volatile compounds also have been reported for identifying the entomological differences of honey [41,42]. Gas Chromatography Mass Spectrometry (GC-MS) is a widely used technique which requires complex sample processing and pretreatment. A well-accepted method for the extraction of volatile compounds prior to separation and identification is Headspace Solid Phase Micro Extraction (HS-SPME). However, this technique requires rigorous testing and optimization of various factors, such as the type of fibers and ionic strength of the extraction medium, to ensure accurate and reliable results. Sharin et al. [43] discriminated Malaysian stingless bee honey according to the different entomological origin (*Heterotrigona bakei*, *Geniotrigona thoracica*, *Tetrigona binghami*) based on volatile compound profiling. Partial Least Square Discriminant Analysis (PLS-DA) model based on physicochemical properties such as pH, moisture, total soluble solid, ash, electric conductivity, and 13 significant volatile compounds, clearly differentiated the honey samples (n = 75) according to three stingless bee species. *T. binghami* honey is marked by significantly higher electrical conductivity, moisture and ash content, and high abundance of 2,6,6-trimethyl-1-cyclohexene-1-carboxaldehyde,2,6,6-trimethyl-1-cyclohexene-1-acetaldehyde and ethyl 2-(5-methyl-5-vinyltetrahydrofuran-2-yl) propan-2-yl carbonate. Copaene was proposed as a chemical marker for *G. thoracica* honey. In this study, prior to GC-MS analysis, chemical methods were carried out to isolate maximum number of potential volatile compounds from the complex honey matrices. One of the challenges of using GC-MS is inconsistency in results due to variations in the complexity in the type of food samples, including honey, and the pretreatment process. On the other hand, Headspace Gas Chromatography Ion mobility Spectrometry (HS-GC-IMS) is a method with no prior complex pretreatment of the sample. Mass spectrometry separates compounds by their mass-to-charge ratio, while Ion Mobility Spectrometry (IMS) is a post-ionization separation technique that separates ions by their size, shape, and charge. Briefly, the separation method works by driving ions through a gas-filled chamber using an electric field, and the ions separate based on their shape, with the more compact ions moving faster than those with an open structure. In a study with lychee honey, 20 from *Apis cerana* and 20 from *Apis mellifera*, from Guangdong province in southern China, the presence of volatiles such as 1-nonanol, phenethyl acetate, and 1-heptanol were significantly higher in *Apis cerana* honey and therefore, can be used as markers for *cerana* honey [42]. Differences of volatile compounds were found between *Apis cerana* and *Apis mellifera* honeycomb [44]. Some scientific studies used beeswax to differentiate the entomological origin of honeybees and stingless bees. Xu et al. [45] demonstrated that the differences between the beeswax of *A. cerana cerana* and *A. mellifera ligustica* were mainly based on the content and composition of hydrocarbons, monoesters, and free acids. After GC-MS/MS analysis of honey from *A. mellifera* and *A. cerana* colonies, Zhang et al. [30] found that Hentriacontane and 17-Pentatriacontene were the characteristic components of *A. mellifera* and *A. cerana*, respectively.

Metabolomics approaches are also used to discriminate honey of different entomological origin. A comprehensive LC-MS based metabolomics approach revealed that 3-amino-2-naphthoic acid and methyl indole-3-acetate are potential markers for *Apis cerana* honey, as these compounds were present in higher amounts in *Apis cerana* honey. On the other hand, kynurenic acid was determined as a marker for *Apis mellifera* honey [46]. In this study [46], Electrospray Ionization Quadrupole Orbitrap High-Resolution Mass Spectrometry was used. ^1^H NMR-based metabolomics approach is also used to authenticate entomological as well as floral origin of honey [47,48,49]. Aqueous (in D_2_O) as well as chloroform extracts of honey were prepared, subjected to ^1^H NMR analysis followed by chemometric analysis Orthogonal Projection to Latent Structure Discrimination Analysis (OPLS-DA) [47]. Unique metabolic fingerprints were obtained for different honey types such as *Apis mellifera*, *Geotrigona-Trigona*, *Melipona*, and *Scaptotrigona* [47]. *Geotrigona-Trigona* honeys contained relatively low quantities of fructose and glucose, and higher contents of di- and tri-saccharides such as raffinose, isomaltose, isomaltotriose, melibiose, and palatinose; *Melipona* pot-honey contained relatively low amount of turanose and of di- and tri-saccharides in comparison to the other honeys. On the other hand, *Apis mellifera* honey was characterized by higher contents of glucose and fructose. Concerning to the minor chemical compounds, *Geotrigona-Trigona* honey differed from other types of honeys in the content of 3-hydroxy-2-butanone and 2,3-butanediol [47]. Schievano et al. [48] used NMR-based metabolomics approach to 67 samples to classify them according to entomological origins i.e., *Apis mellifera*, *Melipona aff. Fuscopilosa*, and *Trigona clavipes*. Another study [49] employed ^1^H NMR Spectroscopy and Ultra-High Performance Liquid Chromatography coupled with Quadrupole Time of Flight Mass Spectrometry (UHPLC-QTOF-MS) followed by chemometrics analysis to classify raw stingless bee honey samples based on entomological origin using. The NMR spectral data indicated presence of d-Fructofuranose in *Heterotrigona itama* honey, β-d-Glucose, d-Xylose, and α-d-Glucose in *Geniotrogona thoracica* honey, and l-Lactic acid, Acetic acid, and l-Alanine in *Trigona apicalis* honey [49]. 

Fourier Transform Infrared (FTIR) spectroscopy is also used for entomological differentiation of honey. FTIR spectral data subjected to PCA showed distinguished clusters for *A. mellifera*, *A.dorsata*, *A. cerana*, and *Trigona* honey samples collected from different provinces of Philippine [50]. FTIR spectroscopy was also applied in order to authenticate honey i.e., differentiation between real and fake honey [51]. The study [51] also depicted that the wavelength range 1600–1700 cm^−1^ was the best to classify the *Apis* spp. and *Tetragonula* spp. honey samples. 

### 2.4. DNA-Based Method

Recently, DNA-based methods are considered as a rapid, accurate, and suitable tool for species identification in animal products and processed foods [52,53,54,55]. This method relies on the identification of insect DNA present within the honey sample, as represented in Figure 1C. It is more precise, reliable, quick, and cost effective for analyzing large sample sizes compared to previously described methods [10]. Most DNA is located in the cell nucleus (nuclear DNA), but a small amount of DNA can also be found in the mitochondria (mitochondrial DNA or mtDNA). The method is based on Polymerase Chain Reaction (PCR) and the different honey samples can be identified according to DNA barcoding or by analyzing the pattern of bands on gel electrophoresis. 

#### 2.4.1. DNA Barcoding 

DNA barcoding is a molecular method that involves amplifying a short region of the target genome through PCR and comparing the resulting sequences with reference sequences available in public databases, such as GenBank, to identify any biological species [56]. It has been widely used for species-level identification and has contributed to the genetic traceability of livestock, agricultural crops, and their food products [56,57]. Recently, DNA barcoding has been developed to identify the entomological origin of honey from *A. dorsata*, *A. mellifera*, *A. cerana*, and *Heterotrigona itama* [58]. Several sets of primers have been designed to amplify the Cytochrome Oxidase I (COI) or 16S rRNA part of mitochondrial DNA (mtDNA) (Table 1). After sequencing, the entomological origin of honey can be identified [58,59]. Furthermore, this Sanger sequencing-based technique has been used to distinguish honey samples derived from different mitotypes (C1 and C2) of the C lineage of *Apis mellifera* based on the sequence of 170 bp obtained from the primer set targeting tRNAleu-COX2 intergenic region of mtDNA [60]. Although this method has been successful in identifying the species of honeybees, it has limitations in identifying the entomological origin of honey in counterfeit mixed honey samples due to the constraints of the Sanger sequencing approach. 

#### 2.4.2. Usage of Species-Specific Primers

Zhang et al. [10] developed a DNA-based method for identifying honey samples from *A. cerana* and *A. mellifera* using two sets of species-specific primers that target MRJP2. The method involves a double PCR followed by gel electrophoresis, which results in an amplicon size of 212 bp for *A. cerana* and 560 bp for *A. mellifera* (Table 2). The specificity of the primer sets also enables the detection of honey fraud using a multiplex PCR, where both sets of primers are used simultaneously in one PCR reaction to detect DNA from both species. This method successfully differentiated honey originated from *A. cerana javana* and *A. mellifera* in Indonesia. However, the authors mentioned that M-F and M-R primer set has to be used with caution since PCR amplicons were observed while using these primers and the DNA extracted from *A. cerana* honey in low annealing temperature [62]. 

Mitochondrial DNA is present in most cells of the organism in relatively high copy numbers. mtDNA is inherited maternally and characterized by a high genetic variation between related species and a low intraspecific variation [64,65,66]. Therefore, it is suitable for taxonomic purposes, such as species identification through DNA barcoding and phylogenetic analysis. Among all mitochondrial genes, COI has shown to be the most interesting and largely used, given its lower mutation rates and high incidence of nucleotide substitution at the third codon position when compared to other protein-coding genes [67,68]. Kim et al. [8] developed two sets of species-specific primers targeting 133 bp and 178 bp of COI gene for *A. mellifera* and one set of primers with amplicon size of 178 bp for amplification of only *A. cerana* DNA (Table 2). Although the sizes of the amplicons are suitable enough for relatively old honey samples as well, Zhang et al. [10] claimed that the *A. mellifera* primer sets are not specific enough for distinguishing of *A. cerana* honey originated from China. Later, Soares et al. [11] designed species-specific primers targeting 111 bp of the tRNAleu-cox2 intergenic region to detect *A. cerana* DNA through PCR. Recently, Mohamadzade Namin et al. [63] developed three sets of species-specific primers targeting NADH Dehydrogenase 2 (ND2) region of mtDNA (Table 2) in order to distinguish entomological origin of major types of honey in Asian market from *A. cerana*, *A. dorsata*, and *A. mellifera*. The results of the specificity test indicated the possibility of differentiation of honey samples based on gel electrophoresis pattern (223, 301, and 376 bp for *A. cerana*, *A. dorsata*, and *A. mellifera*, respectively).

#### 2.4.3. Real-Time PCR-Based Methods

##### High-Resolution Melting Analysis

Quantitative analysis of DNA fragment melt curves after PCR amplification is known as High-Resolution Melt Analysis (HRM Analysis). HRM Analysis is a modern version of amplicon melting analysis. To perform HRM, a real-time PCR detection system with exceptional thermal stability and sensitivity, as well as dedicated software, is required. The use of high-quality qPCR instruments and DNA-binding dyes has enabled the identification of even minor variations in nucleic acid sequences by precisely melting double-stranded PCR amplicons [69]. The species-specific primers developed by Zhang et al. [10] to distinguish *A. cerana* honey and *A. mellifera* honey through gel electrophoresis pattern are also applicable in Real-Time PCR assay through melting curve analysis as a single melt peak for each species was observed (76.4 °C for *A. cerana* and 82.9 °C for *A. mellifera*). In addition, the melting curve analysis demonstrated the possibility of using the primer sets developed by Mohamadzade Namin et al. [63] to identify the entomological origin of honey from *A. cerana* (Tm: 71.9 °C), *A. dorsata* (Tm: 69.2 °C), and *A. mellifera* (Tm: 72.4 °C). Another set of primer was developed by Soares et al. [11] to amplify 140 bp of 16S rRNA gene of the mtDNA to differentiate *A. cerana* and *A. mellifera* honey through high-resolution melting analysis of Real-time PCR. 

This method has been used by Soares et al. [70] to distinguish the entomological origin of honey from different lineages of European honeybee, *A. mellifera*. In their study [70], a two-step method was employed that involved a PCR approach for identifying the A lineage through agarose gel electrophoresis, followed by a real-time PCR that distinguished between the M and C lineages using their high-resolution melting profile. The developed primers targeting 150 bp of the COI gene can be applied to differentiate lineage A, C, and M from various European countries. Since the nucleotide substitutions is the source of the differences between different clusters in the high-resolution melting curve analysis, the sequencing of PCR amplicons can be the alternative method when the Real-Time PCR is not available [71]. 

##### Loop-Mediated Isothermal Amplification (LAMP) Technology

LAMP is another Real-Time PCR (qPCR) technique which has been used to distinguish the entomological origin of honey. Introduced in 2000, LAMP improves the sensitivity and specificity of nucleic acid amplification efficacy [72]. Besides, the ability of LAMP to synthesize DNA using auto-cycling strand displacement activity at a constant temperature (60–65 °C) has rendered expensive thermocyclers and laborious technical optimization of cycling conditions unnecessary and significantly shortened the amplification duration as well [72,73]. As a result of these distinctive characteristics, LAMP has been integrated into the development of diagnostic assays for detecting a wide range of medically significant communicable diseases [74]. Recently, Gao et al. [75] designed specific LAMP primers that target the MRJP2 gene. The developed primers effectively amplify the target gene and successfully distinguish between the *A. cerana* and *A. mellifera* honey with the detection sensitivity of 4 ng/μL and 1 ng/μL for *A. cerana* honey and *A. mellifera* honey, respectively. 

#### 2.4.4. Length Polymorphism of PCR Product 

Variation of the amplicon sizes resulting from the PCR reaction can also be applied as a technique to identify the entomological origin of honey. Utzeri et al. [76] applied a primer set that targeted the tRNAleu-COX2 intergenic mtDNA to identify the lineage origin of European honeybee from which honey samples were collected. The primers produced fragments of 85 bp for *A. mellifera* C lineage (including *A. mellifera ligustica*), 138 bp for M lineage (including *A. mellifera mellifera*), or 152 bp for A lineage (honeybee subspecies of African origin and *A. mellifera siciliana*). These different fragment sizes can be distinguished through a high percentage agarose gel electrophoresis. Honey originated from *A. mellifera iberiensis* showed either 152 bp or 138 bp or both fragment sizes, which is consistent with the hybrid origin of Iberian honeybee populations [76].

#### 2.4.5. DNA Metabarcoding Approach

DNA metabarcoding is a next-generation sequencing approach allowing the identification of the origin of samples with multiple sources [77,78,79]. To prepare the metabarcoding library, two rounds of PCR amplification are needed. The first PCR involves the use of primers with overhanging sequences for amplification, followed by the attachment of a specific tag to the amplicons during the second PCR. These tags serve as a unique identifier for each sample, making it easy to track and distinguish them. The resulting sequences then undergo various bioinformatics processes, including quality control, trimming, merging, dereplication, and chimera checking. The filtered sequences are then compared to reference libraries to facilitate identification. Prosser and Hebert [61] recently developed a metabarcoding-based approach to identify the entomological origin of honey using COI gene (Table 1). This method was successfully applied to distinguish the entomological origin of *A. mellifera* honey and stingless bee, *Melipona beecheii*, honey, and there is perhaps a possibility of using the same primer set to detect the other *Apis* honeybee species. The methodology was assessed using pure honey samples and it would be interesting to assess the possibility of using this method to understand the level of adulteration in the case of mixed honey samples. Further research is required to determine the possibility of using the same primer set to distinguish other *Apis* and non-*Apis* honeybees. One potential limitation of this approach is the possibility of mismatches in the amplification site of phylogenetically distant groups, which could affect its accuracy. Despite these limitations, DNA metabarcoding has significant potential as a tool for identifying the origin of honey samples from different bees.

## 3. Discussion

We have reviewed various methodologies that have been used for identifying the entomological origin of honey. Most previous studies have focused on differentiating honey in specific regions, and further investigation is required to extend the applicability of these methodologies to other geographical locations. 

Although DNA-based methods are considered fast and accurate for entomological origin identification, DNA degradation is one of the most crucial limitations of using these methods for old or processed honey samples [8,76,80,81]. Honey is a complex matrix and the presence of H_2_O_2_ in combination with other components can accelerate the degradation of the DNA remaining inside honey. On the other hand, the acidic pH of honey (ranging from 3.06 to 4.23) [37] depurates DNA and leads to subsequent strand cleavage, which causes dwindling of DNA strands and unsuccessful PCR [82,83,84]. Utzeri et al. [76] also reported that the E2 forward and H2 reverse primers [21], targeting 500–1000 bp of the COI-COII section of the mtDNA, cannot be used in entomological origin authentication analysis due to the lack of amplification. Undoubtedly, shorter amplicons are more accessible using DNA-based methods, however, the age range of honey to which such a methodology can be applied is not completely clear and investigation of the pace of DNA degradation inside honey is crucial. Additionally, designing of the species-specific primers targeting a short fragment of DNA is not possible for all honeybees and stingless bees. However, DNA metabarcoding can be a good solution by which one set of primers can be applied to all honeybee and stingless bee species. As honey from one honeybee species can be stolen by conspecific workers of other colonies or other species [85,86], it is possible that small amounts of honey with a different origin may be present in studied samples. Therefore, the sensitivity of the methodologies should be taken into consideration, and the detection of small amounts of other honey should not be considered honey fraud. It is essential to optimize the methodologies to minimize false positives and false negatives. Quantitative methods that provide the honey content of different species are highly valuable. One option is to analyze the intensity of bands using available software, such as ImageJ, in PCR-based methods following electrophoresis. Additionally, the number of sequence reads provided by metabarcoding analysis can be converted to a percentage and used in quantitative analysis, which requires further investigation. While metabarcoding can sometimes lead to overestimation of some species due to bias in amplification, other advanced methods such as whole-genome shotgun sequencing can provide more accurate quantitative results [87]. These methods should be investigated in future studies.

Proteomics has been utilized in various fields, including food authentication, and has the potential for determining the entomological origin of honey. Previous studies have shown promising results in using protein analysis for honey authentication, and further research could lead to even better results. However, while state-of-the-art technologies can accurately differentiate the origin of honey, they may not be practical for routine testing due to their high cost and time-consuming nature. Therefore, proteomics and genomics may be more appropriate for specific situations, such as when there is a suspicion of honey adulteration or for research purposes.

## 4. Conclusions

The authentication of the entomological origin of honey is an essential part of ensuring the quality, safety, and authenticity of honey for consumers. The detection of honeybee DNA or secretions inside honey allows for the tracing of the entomological origin of honey. Several methods have been developed to authenticate the entomological origin of honey, including protein-based, beeswax-based, and DNA-based analysis. Each of these methods has its strengths and weaknesses and may be more suitable for certain types of honey or specific situations. It is important to have standardized procedures and criteria for the authentication of honey regardless of the methodology used. This can ensure that the results are accurate, reliable, and consistent across different geographical regions. By implementing reliable methods and standards, consumers can be confident that they are purchasing high-quality honey from a trusted source.

## Figures and Tables

**Table 1:** The primer sets which have been applied in the identification of the entomological origin of honey samples through DNA barcoding or metabarcoding approach.

Primer	Primr Sequence	Target Gene	Size of Amplicon (bp)	Method of Discrimination	Reference
COI-300F	GGATTTATTGTCTGAGCACATC	COI	316	DNA barcoding	Kek et al., [58]
COI-300R	TTGCGAATACTGCTCCTATT				
16S-300F	GGACGATAAGACCCTATAGAA	16S rRNA	296–307		
16S-300R	TTGTTAAAAGTCGAACAGAC				
AncientLepF2	ATTRRWRATGATCAARTWTATAAT	COI	120	DNA metabarcoding	Prosser and Hebert, [61].
*Apis*238_R1	TAATCAAAATCTAATATTATTTATTCG				

**Table 2:** Species-specific primer sets for identification of honey samples through PCR or Real-Time PCR.

Primer	Primr Sequence	Target Species	Target Gene	Size of Amplicon (bp)	Method of Discrimination	Reference
M2-F	CATCACTTGAATGATTAAATTTTTTACCA	*A. mellifera*	COI	206	Duplex PCR	Kim et al., [8]
M2-R	TTATTTTTAAATTAATATGAATTAAGTGGGG					
M5-F	CACTTGAATGATTAAATTTTTTACCACCT	*A. mellifera*	COI	133		
M5-R	CTTAAGTTCAATGCACTTATTCTGCC					
C6-F	GGAGGGGGAGATCCAATTTA	*A. cerana*	COI	178		
C5-R	ACCTAATATTGCGTAAATTATACCTAGATTT					
AC1-F	TCTGAATTCAAACTCAAAGTAAAA	*A. cerana*	tRNAleu-cox2	111	PCR	Suares et al., [11]
AC1-R	ATAATATGAGTTTGATTCTTGAAA					
AM1-F	AGCTAATTAAAACAACAATACA	Both species	16S rRNA	140	Melting curve analysis by Real-Time PCR	
AM1-R	AAGGTAGTAAATGTTGAATCATT					
C-F	TTTAACAATAAAAATAATCAGAAGA	*A. cerana*	MRJP2	212	Duplex PCR	Zhang et al., [10]
C-R	TTACATCCTAATTGATTTTAATGCG					
M-F	GCCATCCCTTGAAATTGTCACTCGT	*A. mellifera*	MRJP2	560	Melting curve analysis by Real-Time PCR	
M-R	TCTGCAAACGACCAATCAGGATAT					
AC-F	TCATTAGRTTTTACAAAATCWGATCA	*A. cerana*	NADH2	223	Duplex PCR	Mohamadzade Namin et al., [63]
AC-R	CTTATAACTAAATATGTTAATGATCATA					
AM-F	CYATTAGATTTACTAAAACAGATACT	*A. mellifera*	NADH2	376	Duplex PCR	
AM-R	ATAATTAAATGAATATAAAATAATTATAGCA					
AD-F	TATATTAATTGTTATAACTTACATAAATAA	*A. dorsata*	NADH2	301	Duplex PCR	
AD-R	GGATTAAGAATATATAATATTCATATTTT					

## Data Availability

Data available on request to the authors.
